# Effect of Resin Content on the Structure, Water Resistance and Mechanical Properties of High-Density Bamboo Scrimbers

**DOI:** 10.3390/polym16060797

**Published:** 2024-03-13

**Authors:** Zixuan Yang, Xin Meng, Guangda Zeng, Jinguang Wei, Chuangui Wang, Wenji Yu

**Affiliations:** 1 School of Materials and Chemistry, Anhui Agricultural University, Hefei 230036, China; y2532389280@163.com (Z.Y.);; 2 Key Laboratory of National Forest and Grassland Administration on Wood Quality Improvement & Efficient Utilization, Anhui Agricultural University, Hefei 230036, China; 3 Scrimber Engineering and Technology Research Center of State Forestry and Grassland Administration, Research Institute of Wood Industry, Chinese Academy of Forestry, Beijing 100091, China; chinayuwj@126.com

**Keywords:** bamboo scrimber, resin content, structure, water resistance, mechanical properties, thermal stability

## Abstract

Bamboo scrimber is acknowledged for its eco-friendly potential as a structural material. Its properties are significantly affected by both its density and resin content, but the effect of resin content on the properties under high density is not yet known. In this study, the microstructure, water resistance, mechanical properties, and thermal stability of bamboo scrimbers with varying resin content at a density of 1.30 g/cm^3^ were investigated. The results unearthed that phenolic resin assisted in the densification of bamboo cells during hot pressing, and a higher resin content could effectively reduce the cracks in the scrimber. The inherent cellulose I structure remained unaffected, but an increase in resin content led to a noticeable decline in crystallinity. Additionally, an increase in resin content pronouncedly improved the water resistance and dimensional stability of bamboo scrimbers. The water absorption and thickness swelling were as low as 9.67% and 7.62%, respectively. The modulus of rupture (MOR) exhibited a marginal increase with the amount of resin, whereas the compressive strength and short-beam shearing strength first increased and then decreased. Their peak strengths were 327.87 MPa at a resin content of 15 wt.%, and 168.85 MPa and 25.96 MPa at 11 wt.%, respectively. However, phenolic resin accelerated the thermal decomposition of bamboo scrimbers, and more resin worsened the thermal stability. These research outcomes offer a dual advantage, providing both a theoretical foundation and concrete data that can inform the production and practical application of high-density bamboo scrimbers.

## 1. Introduction

Against the background of global efforts to peak carbon dioxide emissions and achieve carbon neutrality, the spotlight has turned to sustainable alternatives for conventional construction materials. Lignocellulosic composites, praised for their eco-friendliness, renewable sources and the plentiful supply of raw materials, have emerged as a preferred choice over traditional cement, steel and plastics [1]. Among them, bamboo scrimber represents the pinnacle of these sustainable innovations. Produced through laminating long bamboo fiber bundles with artificial resin, the composites possess a dense and robust structure, endowing the scrimber with excellent mechanical properties, water resistance and durability [2,3]. This unique blend of attributes has seen bamboo scrimber gain popularity in a variety of applications, ranging from furniture and decoration to larger-scale construction projects [4]. Thus, bamboo scrimber is increasingly recognized as a viable carbon-negative structural material that both serves infrastructural needs and stewards environmental well-being.

Its properties are largely driven by the changes in density and resin content. Density reflects the mass of bamboo fibers per volume in bamboo scrimber and plays a pivotal role in amplifying the physical and mechanical attributes. Bamboo poles are characterized by their low density, attributed to the hollow nature of the culm and its porous walls. As it is processed into a scrimber, the density sees a dramatic increase of more than 80% [5]. A notable consequence of the increased density is the rise in fiber volume content (FVC), which is directly tied to the improvement in mechanical properties. As the density increases, the FVC undergoes a rise, boosting the mechanical properties [6]. Density also plays a crucial part in augmenting the water resistance and dimensional stability of the bamboo scrimbers [7]. Bamboo’s natural state presents a structure abundant in pores, mainly composed of cell cavities, pits and micropores within the cell wall. These features facilitate the movement and storage of water within the material [8,9]. Coupled with the hydrophilicity of its extractives, cellulose and hemicellulose, bamboo can absorb significant amounts of water, leading to dimensional swelling when exposed to humidity [10,11]. The absorbed water expands the distance between the molecular chains, leading to dimensional swelling of bamboo [12]. The conversion of bamboo into scrimber addresses these challenges by compressing its pores, effectively minimizing the pathways and spaces for water flow and storage. Consequently, the scrimber possesses a markedly reduced potential for water absorption and dimensional swelling. Additionally, the higher density characteristic of the scrimber improves its water resistance and dimensional stability [13].

Resin content is another essential parameter of bamboo scrimber and is a critical determinant of the physical–mechanical properties. In the fabrication of scrimber, phenolic resin is utilized to bond fiber bundles together, with the quality of this bond being pivotal for the resultant mechanical properties [14]. The effectiveness of phenolic resin lies in its ability to penetrate into the cell cavities, creating “glue nails” that securely anchor the cells, thus boosting the material’s mechanical performance [15]. Apart from enhancing mechanical properties, phenolic resin significantly improves water resistance and dimensional stability. Upon curing, the resin forms a barrier with a low moisture diffusion coefficient, which applies a hydrophobic layer on the fiber bundles, safeguarding the bamboo cells against water penetration [16]. Moreover, resin molecules can be deposited within the cell walls, reducing susceptibility to water-induced swelling [17]. Additionally, the interaction between bamboo constituents and phenolic resin reduces hygroscopic sites within the cells, further contributing to dimensional stability [18,19,20]. Research findings underscore the role of resin content in shaping the properties of bamboo scrimbers. Yu et al. [21] observed that although increasing resin content tends to reduce the bending strength of bamboo scrimber, it significantly enhances water resistance and dimensional stability, regardless of the heat treatment methods applied to bamboo bundles. Zhang et al. [22] corroborated that bamboo-based fiber composites with higher resin content exhibit superior adhesive qualities and dimensional stability across different impregnation methods. Consistently, Yu et al. [23] confirmed that both the water resistance and mechanical performance of bamboo-based fiber composites intended for structural applications markedly improve with increasing amounts of adhesive. These studies collectively suggest that the resin content substantially impacts the physical and mechanical properties of bamboo scrimbers, and an increase in resin content is beneficial for improving these properties.

However, the focus in existing research has been predominantly on bamboo scrimbers with densities ranging from 0.80 to 1.20 g/cm^3^, leaving a gap in knowledge regarding those with densities exceeding 1.20 g/cm^3^. The influence of resin content on the properties of bamboo scrimbers with a density greater than 1.20 g/cm^3^ remains unexplored and warrants investigation. In response to this gap, this study introduces a series of bamboo scrimbers at a density of 1.30 g/cm^3^, fabricated by adjusting the dosage of phenolic resin utilized in their preparation. Their microstructure, water resistance, mechanical properties and thermal stability were investigated. The purpose of this study was to evaluate the effect of the resin content on the properties of these high-density bamboo scrimbers. It is anticipated that the findings of this research will furnish theoretical insights and empirical data to guide the production and application of high-density bamboo scrimbers.

## 2. Materials and Methods

### 2.1. Materials

Bamboo fiber mats, characterized by individual fiber thread approximately 1.10 mm in diameter, were sourced from Hongya Bamboo Era Technology Co., Ltd. (Hongya, China). The phenolic resin used in this study was supplied by Dynea Guangdong Co., Ltd. (Zhaoqing, China). It is notable for a solid content of 47.49 wt.%, a viscosity of 41 mPa·s (25 °C) and a pH value of approximately 10. Additionally, all other reagent chemicals required for this research were sourced from Aladdin Biochemical Technology Co., Ltd. (Shanghai, China).

### 2.2. Fabrication of Bamboo Scrimbers

The scrimbers were fabricated following the method outlined in the literature [5]. Initially, bamboo fiber mats underwent an immersion treatment in a dilute phenolic resin solution. The specifics of the immersion duration were tailored to ensure precise resin uptake. Subsequently, these mats were placed in a dryer at 70 °C until the moisture content reached 10%. Following the drying process, the resin-impregnated mats were methodically layered in alignment with the fiber orientation. Hot pressing was carried out at 145 °C for 30 min on a Model 3856 thermo-compressor (CARVER, Muskegon, MI, USA). The process resulted in the production of bamboo scrimber samples, each measuring 300 mm by 200 mm by 20 mm, aiming for a target density of 1.30 g/cm^3^. By adjusting the resin content (i.e., 9 wt.%, 11 wt.%, 13 wt.% and 15 wt.%), a series of bamboo scrimber samples was obtained for comparative analysis. The resultant scrimbers were sanded with 240-grit sandpaper to remove the cured resin on the surface, cut into the required size and conditioned at 20 °C and 65% RH for two weeks for the following experiments.

### 2.3. Structural Characterization

#### 2.3.1. Microstructure Observation

The transverse surface was taken from the central position of the scrimber, and then smoothed with a diamond knife to achieve a pristine surface. Following preparation, the sample was meticulously coated with a thin layer of gold to enhance conductivity and image quality under electron microscopy. The microstructure was thoroughly examined using a VEGA 3 SBH scanning electron microscope (SEM) (Tescan, Brno, Czech Republic). Observations were conducted at an optimized accelerating voltage of 5.0 kV, ensuring detailed visualization of the material’s internal structure.

#### 2.3.2. Crystal Structures of Cellulose in Scrimbers

The samples were meticulously processed into a fine powder with a particle size of 40 μm. Subsequently, X-ray diffraction (XRD) analyses were performed using an XD-6 detector (Persee, Suzhou, China), which was equipped with Cu-Kα radiation. The operations were carried out under the specific conditions of 40 kV voltage and 30 mA current. The diffraction angle (2θ) was comprehensively scanned across a range from 10° to 40°, maintaining a scanning rate of 2°/min. Based on the obtained data, the empirical crystallinity index (CrI) was precisely determined by Equation (1).

CrI = (I_002_ − I_am_) × 100%/I_002_ (1)

where I_002_ is the intensity of the peak at 2θ of about 22°, and I_am_ is the intensity of the baseline around 18.5° [24].

### 2.4. Evaluation of Water Resistance

#### 2.4.1. Water Absorption

Water absorption (*WA*) tests were conducted meticulously according to the Chinese standard GB/T 40247-2021 [25]. The samples, precisely dimensioned to 25 mm × 25 mm × 10 mm and previously dried in an oven, underwent a specific treatment regime to evaluate their water absorption properties. The regime consisted of an initial immersion in boiling water for 4 h, followed by a 20 h stabilization period within a desiccator at 63 ± 3 °C. Subsequently, the samples were re-immersed in boiling water for an additional 4 h. *WA* (%) was calculated by Equation (2).

*WA* = (w_2_ − w_1_) × 100%/w_1_ (2)

where w_1_ (g) and w_2_ (g) were the sample weights before and after treatment, respectively.

#### 2.4.2. Dimensional Swelling

The dimensional swelling test was performed concurrently with the water absorption test, adhering to an identical sample size and treatment protocol as outlined in Section 2.4.1. Length swelling (*LS*, %) was calculated by Equation (3).

*LS* = (t_2_ − t_1_) × 100%/t_1_ (3)

where t_1_ (mm) and t_2_ (mm) were the sample sizes before and after treatment, respectively. In the same vein, measurements were taken for both width swelling (*WS*, %) and thickness swelling (*TS*, %).

### 2.5. Mechanical Tests

#### 2.5.1. Static Bending

The evaluation of static bending properties was carried out employing a three-point bending setup, as delineated in the standard GB/T 40247-2021. The specimen, measuring 150 mm in length, 25 mm in width and 6 mm in thickness, was placed on the supports with a 120 mm gap between them. The loading was applied in a direction parallel to that of the laminating at a constant speed of 5 mm/min, using an AG-50 kN Xplus universal testing machine (Shimadzu, Kyoto, Japan). The MOR (σ_b_ in MPa) and modulus of elasticity (MOE, denoted as *E*_b_ in GPa) were calculated by Equations (4) and (5), respectively.

σ_b_ = (3F_max_ l)/(2bt^2^) (4)

*E*_b_ = l^3^ (F_2_ − F_1_))/[4bt^3^ (a_2_ − a_1_)] (5)

where F_max_ (N) denotes the maximum failure load of the sample, l (mm) represents the length of the span, b (mm) and t (mm) signify the width and thickness of the sample, respectively, F_2_ − F_1_ (N) indicates the load increase within the linear portion of the load-deflection curve, and a_2_ − a_1_ (mm) reflects the increase in deformation between F_2_ and F_1_.

#### 2.5.2. Compressive Test

The compressive test was performed on the AG-50 kN Xplus tester according to GB/T 40247-2021. The dimensions of the specimen were meticulously prepared to measure 10 mm in length, 6 mm in width and 6 mm in thickness. It was subjected to a load along its length at a controlled advancement rate of 3 mm/min. The compressive strength (σ_c_ in MPa) and modulus (*E*_c_ in GPa) were calculated by Equations (6) and (7), respectively.

σ_c_ = F_max_/bt (6)

*E*_c_ = (l × ∆F)/(bt × ∆L) (7)

where F_max_ (N) is the maximum failure load, b (mm) and t (mm) are the width and thickness of the specimen, ΔF (N) is the increase in load in the initial straight-line section of the load–deformation curve, and ΔL (mm) is the increase in deformation corresponding to ΔF.

#### 2.5.3. Short-Beam Shearing Test

The shearing tests were carried out using the AG-50 kN Xplus tester in strict accordance with the GB/T 40247-2021 standard. The dimensions of the specimen prepared for testing were 60 mm in length, 25 mm in width and 6 mm in thickness, with a designated span of 40 mm between the two roller supports. The loading was applied parallel to the laminating direction of the sample at a speed of 5 mm/min, continuing until the sample fractured. The shearing strength (τ in MPa) was calculated by Equation (8).

τ = (3F_max_)/4bt (8)

where F_max_ (MPa) is the maximum failure load, and b (mm) and t (mm) are the width and thickness of the specimen.

### 2.6. Thermogravimetric Analysis (TGA)

TGA was conducted under a nitrogen atmosphere on an SDT 650 thermal analyzer (TA Instruments, New Castle, DE, USA) designed to analyze the pyrolysis process of bamboo scrimbers. For the analysis, approximately 10 mg of finely powdered scrimber was placed into a platinum crucible. The sample was then uniformly heated, starting from room temperature and gradually reaching up to 800 °C at a steady rate of 10 °C/min.

## 3. Results and Discussion

### 3.1. Microstructure

**Figure 1:**
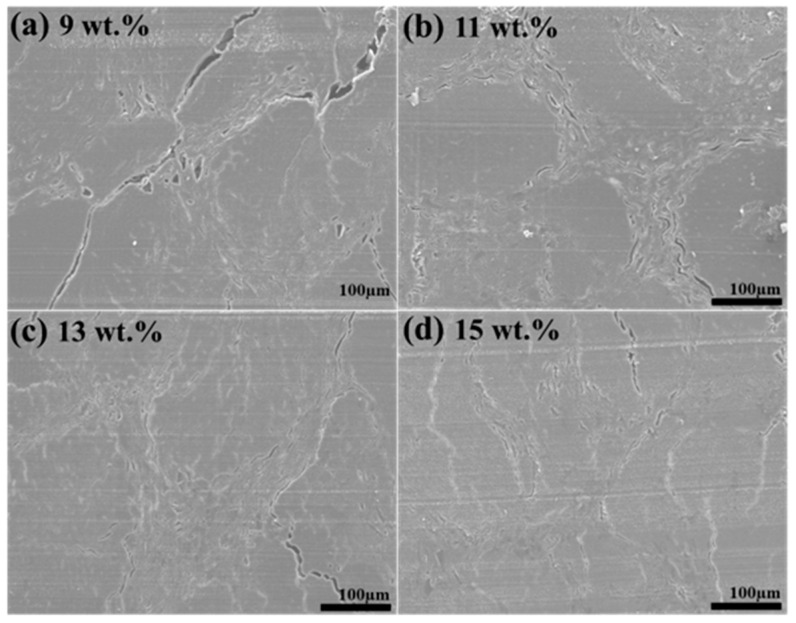
Microstructure of bamboo scrimbers with different resin contents.

Bamboo has a porous structure, featuring vascular bundles entwined within a matrix of parenchyma cells [26]. Within these bundles, fiber cells surround the vessel cells. As the bamboo was processed into a scrimber, the porous structure was significantly densified. As illustrated in Figure 1, parenchyma cells were observed to collapse layer by layer along the cross-sectional view. Nevertheless, the sample still retained some cell cavities. The fiber bundles displayed a distinct outline, with the fiber cells largely preserving their original appearance. When the resin content was 11 wt.%, the parenchyma cells were closely connected to the fibrous cells, achieving nearly complete integration. A few parenchyma cells, however, were scattered around the fiber bundles. Elevating the resin content to 13 wt.% resulted in a total amalgamation of parenchyma cells and fiber cells, where larger fiber cells with significant cavities were fully compacted. With a further increase in resin content to 15 wt.%, the cellular structure becomes entirely congested, leaving only isolated pores visible. These changes could be attributed to the “softening” effect of phenolic resin, which prominently reduces the Young’s modulus of woody cell walls [27]. Therefore, the cell walls exhibited increased compressibility when exposed to phenolic resin. At the low resin content, the cell wall was insufficiently softened, leading to the formation of some cracks. However, as the resin content reached a certain threshold, the cells became more compressible, minimizing crack formation. Therefore, a higher amount of phenolic resin is effective in mitigating cell wall damage during the hot pressing process.

### 3.2. XRD Analysis

**Figure 2:**
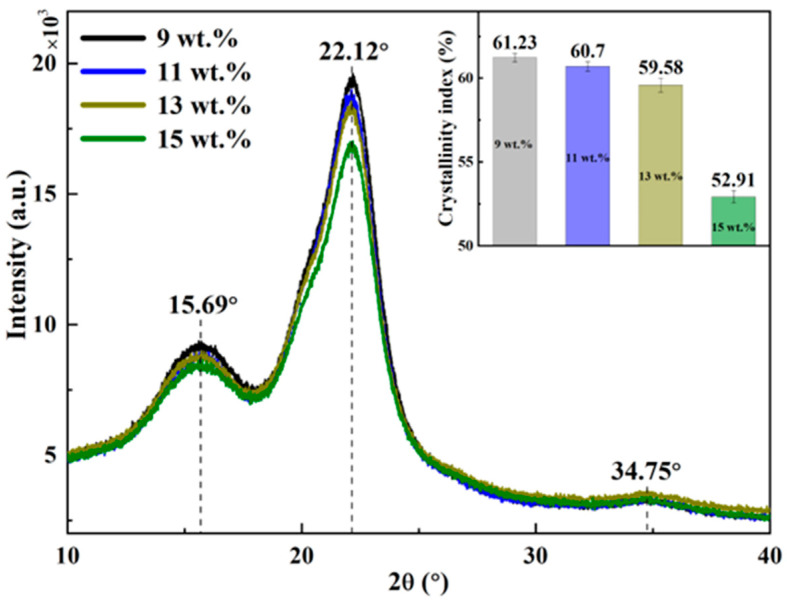
XRD patterns of bamboo scrimbers with varying resin contents.

The XRD patterns of the bamboo scrimbers are presented in Figure 2. Two prominent peaks appear at 15.70° and 22.17°, and a weak peak occurs at 34.75°. The peak at 15.70° comprises two sub-peaks around 14.8° and 16.50°, which correspond to the (1$\bar{1}$0) and (110) planes, respectively [28]. Meanwhile, the peak at 22.17° is identified with the (200) plane, and the subtle peak observed is ascribed to the (004) plane [29]. These peaks are characteristic of the typical cellulose I structure, indicating that the bamboo scrimber maintained the native cellulose structure found in raw bamboo.

However, the crystallinity index exhibited variability in association with the resin content. Illustrative data presented in the inset reveal that the crystallinity index at a resin content of 9 wt.% was recorded at 61.23%. When more resin was added to the scrimber, the index showed a decreasing tendency. Specifically, when the resin content was elevated to 15 wt.%, the crystallinity index diminished to 52.92%, marking a reduction of approximately 14% from the index at a resin content of 9 wt.%. This decrease could be due to chemical reactions between the hydrogen groups within the crystalline regions of bamboo and the phenolic resin, which likely increase the proportion of amorphous cellulose in the scrimber [30].

### 3.3. Water Resistance

**Figure 3:**
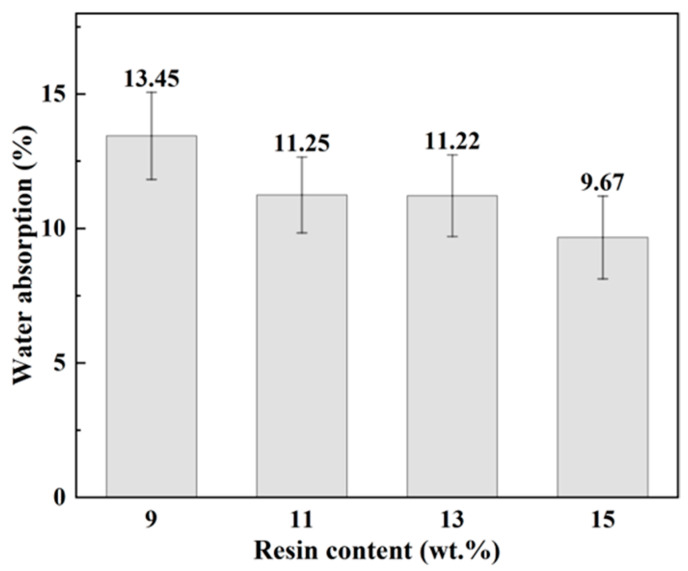
Water absorption of bamboo scrimbers with varying resin contents.

The water absorption of bamboo scrimbers with varying resin contents is illustrated in Figure 3. The water absorption stood at 13.45% with a resin content of 9 wt.%. It exhibited a gradual decrease as the resin content increased. Notably, as the resin content increased to 15 wt.%, the water absorption dropped to 9.67 wt.%, which is 28% less than at a resin content of 9 wt.%. This trend underscores the effectiveness of phenolic resin in reducing water absorption. The resin undergoes curing within the cellular structure of the bamboo, forming a hydrophobic layer that protects the cells from water invasion [31]. Additionally, the interaction between phenolic resin and bamboo components diminishes the presence of hydrophobic groups within the cells [32]. Consequently, the utilization of greater amounts of resin in the scrimber improves its water resistance, leading to a reduction in water absorption as the resin content rises. Therefore, augmenting the resin content in bamboo scrimbers results in superior water resistance.

### 3.4. Dimensional Stability

**Figure 4:**
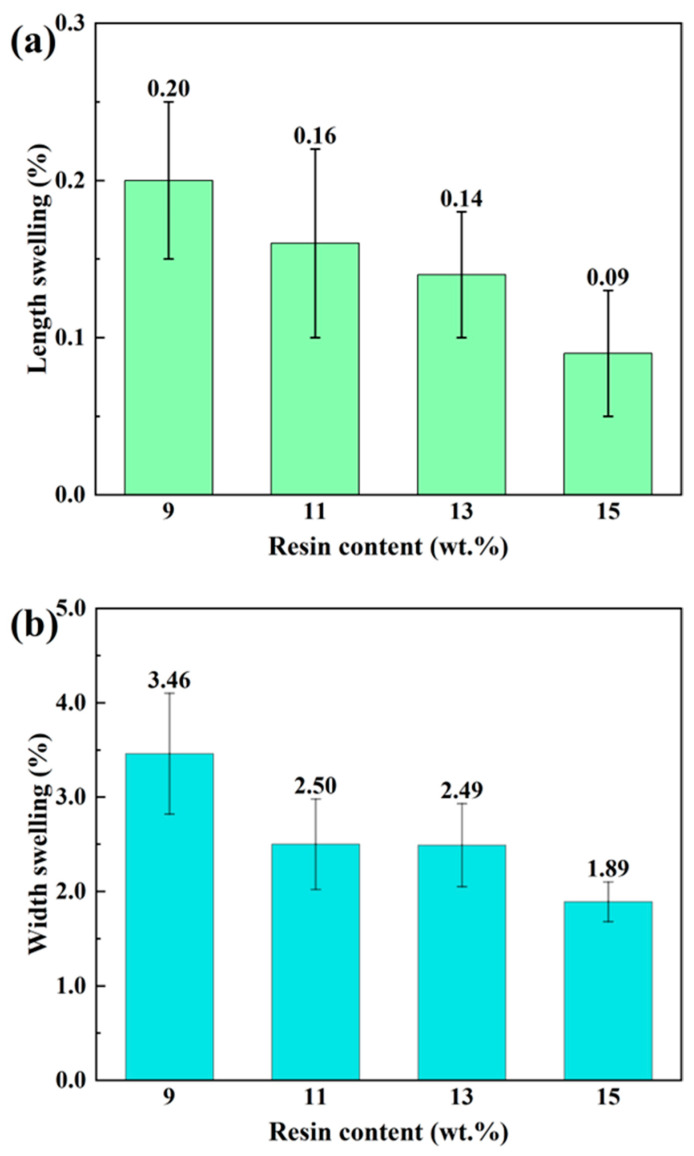

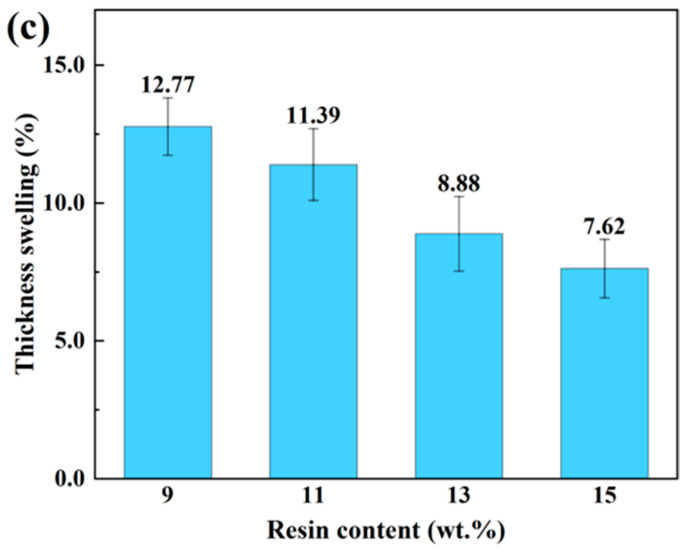
(**a**) Length swelling, (**b**) width swelling and (**c**) thickness swelling of bamboo scrimbers with different resin contents.

The absorbed water gave rise to dimensional swelling. The dimensional swelling across the length, thickness and width of bamboo scrimbers with varying resin contents is shown in Figure 4. A notable reduction in dimensional swelling is observed with increasing resin content. Specifically, for scrimbers with a resin content of 15 wt.%, the swelling rates were recorded as 0.09% for length, 1.89% for thickness and 7.62% for width. These findings highlight the pivotal role of phenolic resin in enhancing dimensional stability. Beyond its efficacy in curbing water absorption, the resin is deposited extensively within the cell walls, effectively mitigating the swelling response following water ingress [18].

The dimensional swelling of bamboo scrimbers across length, width and thickness exhibits distinct patterns: length swelling is less than width swelling, which in turn is less than thickness swelling. For example, at a resin content of 13 wt.%, the length swelling was 93.57% lower than the width swelling and 98.42% lower than the thickness swelling. The differences can be attributed to the varying degrees of cell wall deformation in each direction during hot pressing. Specifically, bamboo cells mainly deformed along the thickness direction, with relatively less deformation occurring in the width direction. Deformation along the length direction was minimal by comparison. As a result, the observed swelling was greatest in the thickness direction, moderate in width, and least in length. The differential swelling underscores the impact of cell wall deformation on the dimensional stability of bamboo scrimbers, influenced by the resin content.

### 3.5. Mechanical Properties

**Figure 5:**
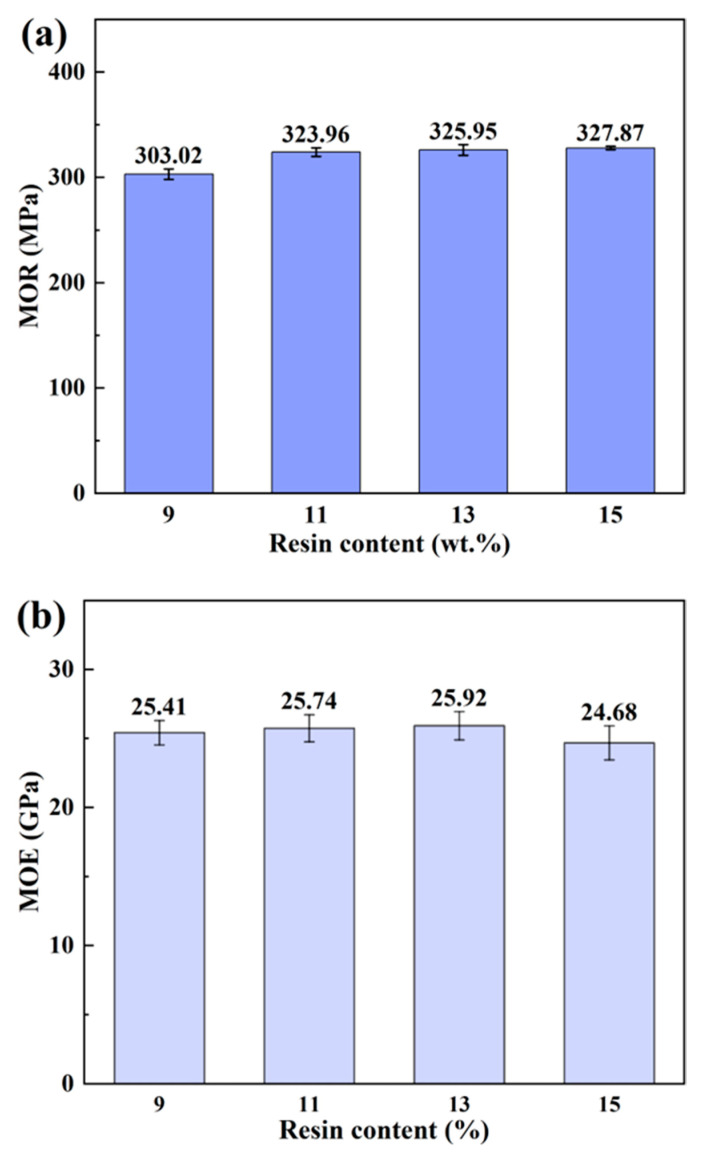
(**a**) MOR and (**b**) MOE of bamboo scrimbers with different resin contents.

The static bending properties of bamboo scrimbers with varying resin content are shown in Figure 5, illustrating a nuanced relationship between resin content and mechanical properties. The MOR exhibited a marginal increase with higher resin content. For scrimbers with a resin content of 9 wt.%, the MOR was measured at 303.02 MPa. An increase of approximately 7% in the MOR was noted as the resin content was adjusted to 11 wt.%. A further 0.6% increase was observed when the resin content was brought up to 13 wt.%. However, increasing the resin content beyond 13 wt.% resulted in a slight decrease in the MOR. In fiber-reinforced composites, the flexural property is implicated in FVC [33]. For the scrimbers, FVC was consistent, and thus the MOR barely changed. The MOE, much like the MOR, followed a similar pattern with changes in resin content ranging from 9 wt.% to 13 wt.%. This trend can be traced back to the introduction of low-molecule-weight phenolic resin. By incorporating a specific phenolic resin into bamboo tissues, cell walls were improved dramatically in strength and stiffness, especially for parenchyma cells [15]. However, past a certain threshold, the advantage of adding more resin diminishes. This is partly due to the innate low modulus of the phenolic resin and its effect on the crystallinity of the bamboo [24,34]. Since the MOE for woody materials is correlated with the crystallinity index, a decrease in crystallinity, induced by excessive resin, can lead to a dip in the MOE [35]. When the content exceeds 13 wt.%, there is an implication that the surplus of phenolic resin might inadvertently reduce the overall improvement in the MOE.

**Figure 6:**
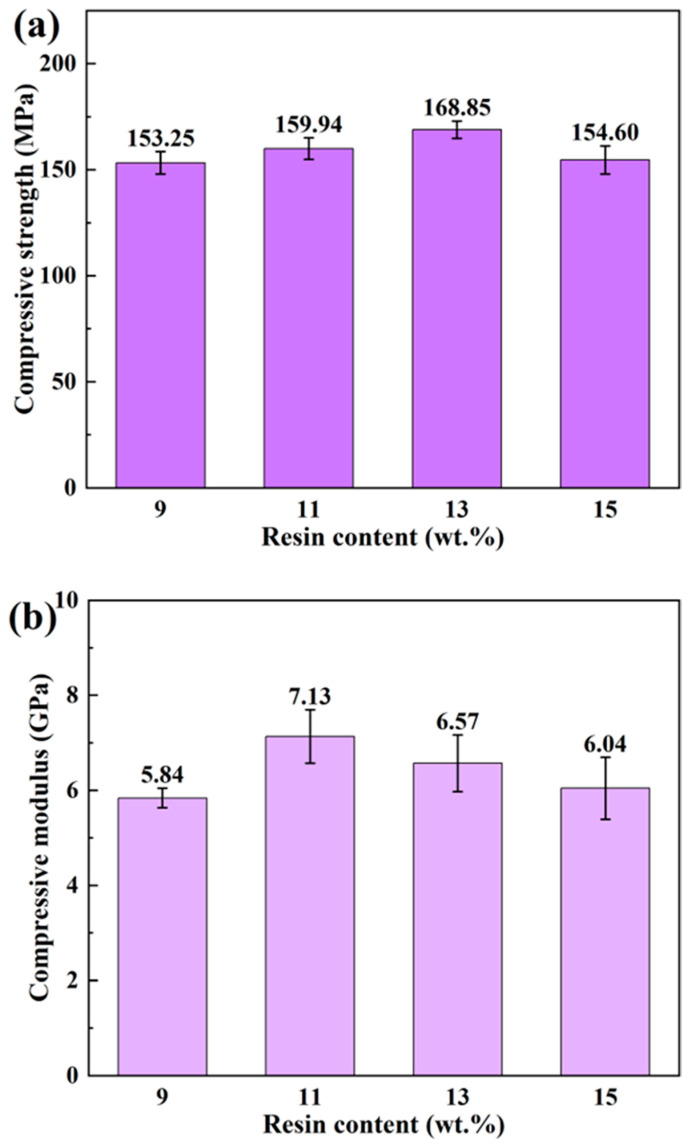
(**a**) Compressive strength and (**b**) modulus of bamboo scrimbers with different resin contents.

The compressive properties of bamboo scrimbers, as influenced by different resin contents, are depicted in Figure 6. With increasing resin content, the compressive strength of the scrimbers improved, reaching a peak at 168.85 MPa at a resin content of 13 wt.% before it began to decline. A similar trend was found in the compressive modulus, which ascended to a zenith of 7.13 GPa at a resin content of 11 wt.%, then decreased as the resin content continued to rise. These trends suggest a correlation with the bonding properties of bamboo fiber bundles. At the adhesive interface of bamboo fibers and phenolic resin, mechanical bonding mechanisms such as hooking, anchoring and stapling play a crucial role [15]. More resin added to the scrimber typically improves the bonding quality and, by extension, its compressive properties. However, beyond a certain threshold, excessive resin can compromise the bonding efficacy, leading to a decrease in compressive properties. This observation is corroborated by the outcomes of the short-beam shearing tests.

**Figure 7:**
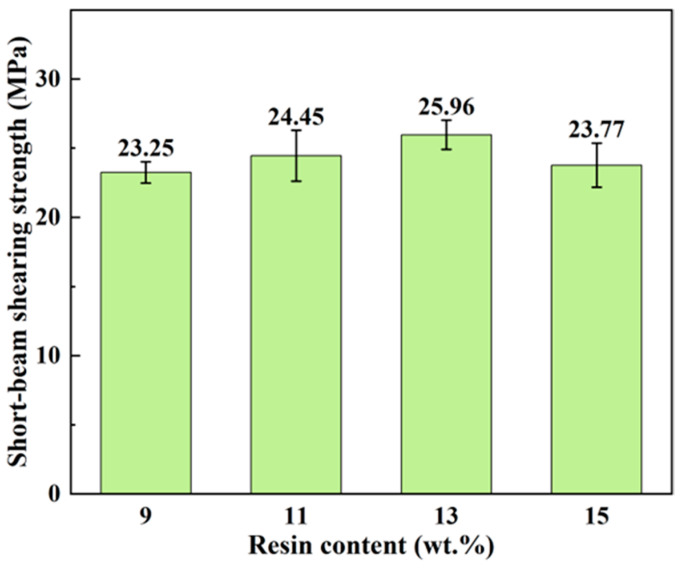
Short-beam shearing strengths of bamboo scrimbers with different resin contents.

Figure 7 presents the variations in short-beam shearing strength among bamboo scrimbers with different levels of resin content. Similar to the trends observed in compressive strength, the shearing strength initially increased before declining as more resin was used in the scrimber. The maximum strength, amounting to 22.37 MPa, occurred at a resin content of 13 wt.%. The short-beam shearing strength serves as an indicator of the bonding quality of laminated woody panels [36]. The higher strength indicates a better bonding quality between the bamboo fibers and phenolic resin. What is more, more resin infiltrating into cell walls further strengthens the cells, leading to a notable improvement in shearing strength. However, the decrease in shearing strength as the resin content exceeded 13 wt.% suggested that excessive resin was detrimental to bonding bamboo bundles. Therefore, an appropriate resin content is good for optimizing the compressive and shearing properties of high-density bamboo scrimbers.

### 3.6. Thermal Property

**Figure 8:**
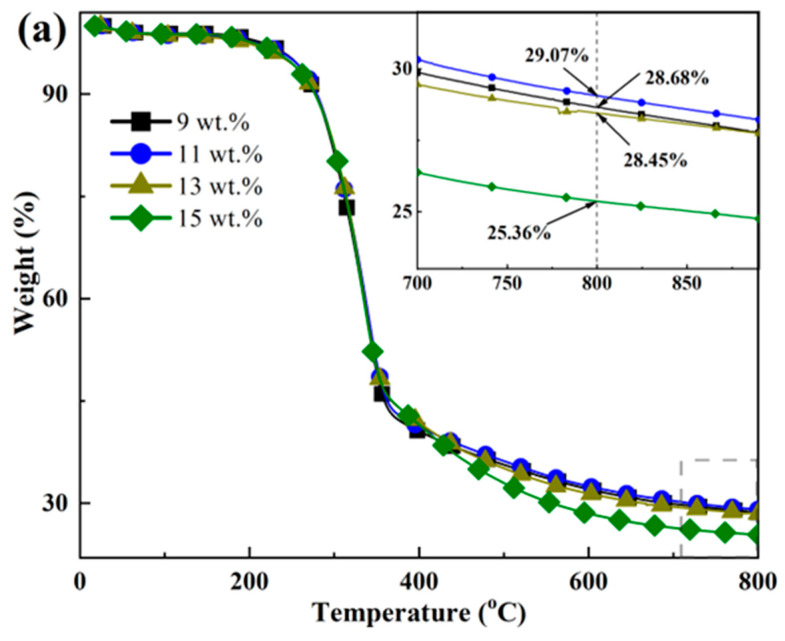

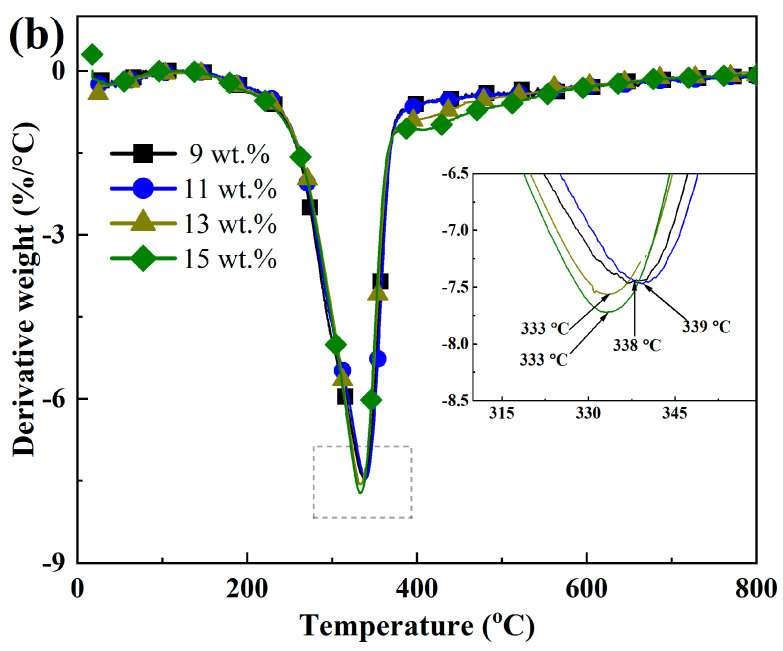
(**a**) TG and (**b**) DTG curves of bamboo scrimbers with different resin contents.

The TG and DTG curves for bamboo scrimbers, as depicted in Figure 8, exhibit similar evolution, which can be typically categorized into three distinct stages. The initial stage, occurring at temperatures below 150 °C, is characterized by a slight weight loss ranging from 1.13% to 1.66% due to the evaporation of water. In the subsequent stage, spanning temperatures between 150 °C and 405 °C, there is a significant decomposition of the bamboo scrimbers. The initial mass was lost by approximately 60%. The final stage, manifesting at temperatures exceeding 600 °C, shows the near-complete decomposition of the scrimber.

The peak temperature was around 335 °C. According to the report by Yang et al. [37], hemicellulose decomposition is noted within a range of 220 °C to 315 °C, cellulose pyrolysis occurs between 315 and 400 °C, and lignin undergoes pyrolysis across a broad spectrum of temperatures from 150 up to 800 °C. This clarifies that the pronounced peak observed in the bamboo scrimbers’ thermogram is primarily due to cellulose pyrolysis, while the shoulder peak around 330 °C is believed to result from the overlapping decomposition of both hemicellulose and lignin. Interestingly, the pyrolysis peaks of the scrimber shift to low temperatures with an increase in resin content, suggesting that phenolic resin contributes to a decrease in the decomposition temperatures of bamboo components. Furthermore, the data reveal that a higher resin content leads to a lower residual yield at 800 °C, which implies that adding more resin diminishes the thermal stability of the bamboo scrimbers.

## 4. Conclusions

In this study, high-density bamboo scrimbers were developed using various levels of phenolic resin to evaluate the effect of resin content on their microstructure, water resistance, mechanical properties and thermal stability. The investigation revealed that the bamboo cells were severely compressed during the hot pressing process, with increased resin application leading to fewer cracks. The structure of cellulose I remained intact, but crystallinity decreased as the resin content grew, reaching a nadir at 52.91%. Enhanced resin amounts notably augmented the water resistance and dimensional steadiness of the scrimbers. Notably, when the resin content was 15 wt.%, the metrics for water absorption and length, width and thickness swelling stood at 9.67%, 0.09%, 1.89% and 7.62%, respectively. Moreover, the resin content showed a remarkable effect on the mechanical properties. The MOR incrementally rose with the resin content, peaking at 327.87 MPa. The compressive and short-beam shearing properties exhibited a parabolic trend, achieving their apexes at a resin content of 11 wt.%, with values of 168.85 MPa and 25.96 MPa, respectively. Additionally, an increase in resin led to an observed shift in the decomposition peak towards lower temperatures and a heightened mass loss at the latter stages of thermal decomposition. This suggests that the higher resin contents detract from the scrimbers’ thermal robustness. These research findings have the potential to serve as both a theoretical basis and as practical insights facilitating the optimization of high-density bamboo scrimber production and application processes.

## Data Availability

The data used to support the findings of this study are available from the corresponding author (weijorn@163.com) upon reasonable request.
